# Book Reviews

**Published:** 1820-11

**Authors:** 


### CRITICAL ANALYSIS

OF

### RECENT PUBLICATIONS, IN THE DIFFERENT BRANCHES OF MEDICINE AND SURGERY;

### SELECT MEMOIRS, AND HISTORIES OF CASES;

### In the Literature of Foreign Nations.

---

Πατρίδες ἄρα
Ανδράσιν, ὦ πάτραις ἄνδρες, ἀγαλλόμεθα.

---

*Ueber lebende Würmer im lebenden Menschen.* Von Dr. Bremser. 4to. seit. 284. Schaumburg und Comp. Wien, 1819.—i. e. *On living Worms in living Men, &c.*

THIS work, as we stated in our review of that of Professor Rudolphi, is intended expressly for the instruction of physicians; and we shall, in the analysis we are about to give of it, confine ourselves very closely to those parts of it which are most intimately connected with this object, and shall consequently pass over several points in the history and physiology of the entozoa that are adventitious to this end, or that were noticed by us in the review just mentioned.

A preface to this treatise is occupied with some remarks on the history of helminthology, and an exposition of the motives which induced the author to publish his observations and opinions. He shows, that but little knowledge of the class of animals under consideration was obtained previously to Zeder and Rudolphi; and he pays a just tribute of praise to the latter of those naturalists, and to the *Lezioni* of Professor Brera, which we have before us for the purpose of selecting from them, in the course of this article, some observations which we think will be of utility to the medical practitioner. The author also tells us here, with much frankness and simplicity, that he has acquired much celebrity for the treatment of patients affected with worms; and that he has annually seventy or eighty persons of this kind under his care.

The first chapter is devoted to a disquisition on the origin and formation of entozoa in man and other animals. We gave the sum of the author's opinions on this point in our review of the work of Rudolphi. Fifty pages would hardly comprise a fair exposition of the essential arguments that may be adduced in favour of the different views that have been taken of this much-disputed question; and we think our Journal can be occupied in a better manner than by such a discussion. Indeed, all that has been said hitherto on the subject, *proves nothing*. It is a very unsatisfactory conclusion of the arguments in favour of the spontaneous generation of entozoa, that, in the words of Dr. Bremser, "they are engendered spontaneously, and as simple products of organic matter, living in perpetuity, and endowed throughout with the property of a tendency to form one whole being in itself." When a due degree of the closeness and profundity of reasoning, and of the acute and comprehensive views, manifested in the second and third chapters of the first book of the *General Indications* of Daniel Pring, shall be employed in the discussion of this subject, then, and not till then, may we expect to find some inferences produced that will be worthy of serious consideration; and to see expelled from medical writings such ingenious obscurities as those of Treviranus and his disciples.

The second chapter treats of the systematic arrangement of entozoa in general, and presents a history of the several classifications which have hitherto been proposed. The author thinks that of Rudolphi the best, though it requires some modifications; and he considers that, in a medical point of view, it is advisable to regard them as they exist in the *intestines* or in *other parts* of the human body.

A description of the worms which inhabit the intestines of man, constitutes the third chapter. Those considered by the author are the following.

The *tricocephalus dispar;* or, as the author terms it in his own language, *peitschenwurm* (lash, or whip worm), or the *haarkopf* (capillary-head). The naturalist's description is, *parte capillari longissima, capite acuto indistincto, corpore maris spiraliter involuto, feminæ subrecto.*

It is most frequently found in the coecum. Wagner and Block state that they have never found it but in this part; but Werner says he has seen it in the ileum. Its length is from one inch and a half to two inches, two-thirds of which is fine and filiform. It is white when not tinged with coloured matter present in the alimentary canal. The filiform portion is the anterior part of the worm. The male is somewhat smaller than the female. The mouth, in the fine anterior extremity, is hardly perceptible; it is a cross mark, from which extends, in a straight direction, the alimentary canal, and which passes into the thicker end. This portion of the animal is twisted, in the male, into a spiral form, and in this are situate the spiral seminal vessels, originating from a small pellucid tube, and terminating in the projecting genital organ. The female has a longer filiform portion than the male. The ovaries, containing ova of an elliptic figure, are situate around the alimentary canal in the thick portion of the animal, which is a little crooked and flattened, but not turned into such a spiral form as that of the male. A small opening at the extremity appears to serve both for anus and vagina.

Roederer was supposed to have first observed this worm in the

Anatomical Theatre at Gottingen in 1760; but it had been previously noticed by MORGAGNI, (*Epist.* xiv. 42.) Later researches have led to a belief that it exists only in dead bodies, and here there are sometimes a thousand or more present in the same subject. Professor BRERA states that no instance is on record of the expulsion of it from the living body. The same genus has also been witnessed in apes, dogs, and many of the *glires* and hoofed animals. Its filiform extremity is ordinarily found to be firmly fixed to the inner surface of the intestine; a circumstance which favours the opinions of PALLAS and some later authors, of this extremity being the anterior part of the animal; though it was supposed to be the tail by former writers.

The *oxyuris vermicularis;* called by Dr. Bremser the *pfriemenschwanz* (spike-tail). Its description for the naturalist is, *Capitis obtusi membrana laterali utrinque vesiculari, cauda maris spirali obtusa, feminæ subulata recta.*

This worm is commonly known by the name of *ascaris vermicularis.* Its seat is in the large intestines, and especially in the rectum. WOLF says he found a considerable number of them in a small pouch between the coats of the stomach. They usually exist in groups, rolled together into masses. They sometimes exist in the vagina of women, producing there very troublesome irritation: it is probable that they are not originally developed there, but have passed into it from the rectum by the anus. The male and female differ in regard to form, though they both equally vary in length from four or five lines to an inch. The anterior part of the male is surrounded by a vesicular, pellucid, membrane, within which is seen the œsophagus; which, until it terminates in the globular stomach, is of the figure of a pestle. The intestinal canal, from the termination of the œsophagus, becomes gradually larger, and turns, at the extremity, in a spiral form. The seminal vessels are situate around the intestinal canal. The ovaries in the female have the same relative situation. The female, about the head and for the first third of the length of the worm, is somewhat thick it then becomes suddenly more slender, and runs into a spike-like tail, the extremity of which is so fine that it can hardly be perceived by the eye. This is one of the most abundant and most common of the intestinal worms of man. GOETZE supposed that it produced living young; but the author says this notion is erroneous. This is the species of worm which Dr. BARRY, of Cork, supposed he had discovered in a well near Macroom in Ireland, and in the intestines of some persons who drank the water of that well. But it is to be regretted that Dr. Barry did not examine the worms alluded to with sufficient minuteness, to show in a satisfactory manner the identity of nature of those in the well and those in the intestines of the persons in question, and whether or not they were really *ascarides vermiculares.*

The *ascaris lumbricoides;* the *spulwurm* (bobbin-worm). *Corpore utrinque sulcato, cauda obtusiuscula.*

This worm is found in the small intestines, not only of man, but also of oxen, pigs, and horses. It is ordinarily from six to ten lines in circumference, and from six to ten, or even fifteen, inches in length. It is but very seldom less than an inch and a half in length.

Its colour is a reddish-brown; but this is lighter or darker as the colour of the food present in the alimentary canal of the animal varies: sometimes it is of a bluish-red hue. The generative organs can, for the most part, be discerned through the common integuments; and between them the intestinal canal is distinguishable by its brownish colour. The head is clearly marked out from the body by a circular depression. There are on the head three protuberances, furnished with valvular orifices, serving as mouths, and from which tubes can be traced extending to the intestines. Those protuberances can be thrust out or retracted by the animal, as Goetze remarked when he discovered one sucking by their valvular openings. The whole three eminences are, also, capable of being approached together, so as to form a sharp conical point at the extremity of the worm, or a sort of mouth with triple jaws. The male is smaller than the female, and has a crooked and slender tail, from which, within a few lines of its extremity, the genital organ is sometimes seen projecting. The voluminous generative organs of the female fill almost the whole of the posterior part of the animal: the tail is here straight. This worm lays eggs, and does not bring forth living young, as Wendelstadt and some others believed. There is a small opening, about two inches from the head in a worm of moderate size, which is the aperture of the vagina, from one-third to half an inch in length, and which terminates in the bifurcated uterus. The double uterus continues to run backwards from the vagina, and each horn of it terminates in a slender duct which forms a multitude of convolutions, and which are reverted, in a winding manner, about the uterus. These fine convolutions ordinarily contain a whitish tenacious fluid, and the uterus itself a multitude of ova. It is these convolutions, or the spermatic vessels of the male, which, as Tyson proved, have been mistaken for living young ones in this species of worm.

They commonly exist in great numbers together in the human intestines, and in persons of adult age as well as in children, though in the latter much the most frequently. They are not uncommonly found with other worms. Rosenstein mentions the case of a child who voided at once ten worms of this species, several ascarides vermiculares, and a portion of a tænia several feet in length.

The *botriocephalus latus*, or *tænia lata; bandwurm* (tape-worm). Nat. descrip. *Capite foveisque marginalibus oblongis, collo subnullo, articulis anterioribus rugæformibus, insequentibus plurimis brevibus subquadratis latioribus, ultimis longiusculis.*

Its seat is the small intestines. It is commonly thinner, and often broader, than the *tænia solium,* with which it has been confounded. The ordinary length of it is about twenty feet; its greatest breadth is seldom less than six lines, and it is sometimes one inch. It is ordinarily whitish in colour; but, when the worm is kept in spirits of wine, it becomes grey or somewhat brown. It has two longitudinal apertures at the sides of its head; but neither of these, in the opinion of the author, is the organ by which it receives its food: the proper mouth, according to Dr. Bremser, is situate in the middle between those openings. The neck and commencement of the body are very slender, and are, as well as the whole body, formed of numerous joints, which it is not necessary to describe in a minute manner, they are so familiar to every medical practitioner. The joints of the beginning of the latter part are nearly square, whilst those of the body are much greater in width than in length. The tail is obtuse, and as it were truncated. Each lateral margin of each joint has an aperture, belonging to the organs of generation, as stated in the review of the work of Rudolphi. Two canals also run longitudinally through the whole of the animal, near its lateral margins, and are supposed by Rudolphi to be nutrient vessels: they terminate in papillæ situate on the head. There is also a middle canal, extending from the opening in the centre of the head throughout the whole length of the animal. Pallas and Goetze doubted whether this central canal of one ring was continuous with that of the contiguous rings, as they could not pass an injection through them from one to the other; but Winslow stated that he had succeeded in this object. This is supposed by Dr. Bremser to be the proper alimentary canal.

The *tænia solium*, or *tænia cucurbitina; kettenwurm* (chain-worm). Nat. des. *Capite subhemisphærico, discreto; rostello obtuso; collo antrorsum increscente, articulisque anticis brevissimis, insequentibus subquadratis, reliquis oblongis, omnibus obtusiusculis, foraminibus marginalibus vage alternis.*

It is found in the small intestines of all European nations; it is known also to be abundant in those of the Egyptians. It is peculiar to man, and is rather common amongst adult persons. Rudolphi says that Dr. Bremser was the first person who distinguished it from the *tænia lata*. It has sometimes been termed the *armed tænia*, (that last described being called the unarmed tænia,) and not unfrequently the *solitary worm*, because it has been supposed, erroneously, that only one existed at a time in the intestines. A perfect, full-grown worm of this kind, has not yet, it would appear, been seen. Ordinarily, before the anterior part is fully developed, the posterior, having become full of mature ova, separates from it. The size of this worm is therefore unknown. Portions of from twenty to twenty-four feet in length have not unfrequently been found in men. In those cases where several pieces, making up together a much greater length, have been evacuated, there has, the author thinks, either been more than one worm present, or the pieces have been passed at different times, after some considerable intervals. The breadth of it near the head is often hardly equal to one-half or one-third of a line. The thickness is very variable: sometimes it is very thin and almost pellucid. The head is ordinarily very small, but generally sufficiently large to be seen distinctly by the eye, unassisted by a lens, in the dead animal; but in the living state it is undistinguishably continuous with the neck, and the two parts are congruous in their motions. From this head there arise four tubular or papillous mouths, from each of which a canal extends throughout the whole length of the worm; and these canals are supposed, by the generality of naturalists, to be alimentary tubes. Between those mouths, from the whole circumference of the extremity of the head, there arises a prominent circle, forming a sort of ring, in which is fixed a double row of small crotchets; and in the centre of this ring there is a very small opening, which is the origin of a canal which traverses the centre of the worm, like that of the middle canal of the tape-worm: this canal contains a fluid constituted of a globular and albuminous matter. The neck is very slender, and the joints very small. When the animal is young, the joints of the body are almost square; and, as the animal becomes older, they increase more in length than in breadth, so that at last each joint forms a parallelogram with its greatest diameter longitudinally: this diameter is sometimes equal to half an inch. In the margins of the more perfect and fully-developed joints we perceive, alternately right and left, small papillous protuberances, having an opening in the centre which leads to the ovaries; and the ova may be pressed from them. This, as well as the worm last described, is hermaphrodite. Werner discovered the existence of the organs of generation of the two sexes in each ring or joint. He states that two canals open into the marginal papillæ of each ring: the upper one ending in a tubercle, which he considered to be the male organ; and the inferior one being filled with ova.

The author states that he has frequently found two or three of these worms existing together in the intestines; and M. Broussais (vol. xxxiii. p. 7. of this Journal), mentions having found eight *tæniæ* in the dead body of a soldier, but he does not designate the species: that is to say, whether they were the tæniæ *latæ* or *cucurbitinæ*. The latter is the most common in Europeans in general, though the former is said to be most frequent in Prussia, Poland, and in Switzerland. Like other worms of the same genus, the number of joints of the chain-worm increases as the animal becomes older. The author does not acknowledge that separated posterior joints will form into a new entire worm; but such joints may contain ova, which may be expelled and become developed. It is generally supposed that the head, with a few of the joints of the body attached to it, will continue to grow and develop new joints. When portions of the worm are expelled without a head, the author recommends that the latter part should be sought for distinctly in the feces, in a manner similar to that which Dr. Powell (in the sixth volume of the Transactions of the College of Physicians), recommends for the discovery of gall-stones: that is, by pouring water on them, and stirring them together, and then carefully examining the fluid.

Those above described are the only species of worms which ordinarily inhabit the intestinal canal of man. The numerous diversities of appearance in several that have been observed by different writers, are all referable to *varieties* of the foregoing as *species*, in the terms of the naturalist. Several animalculæ of a very different and extraordinary kind have been evacuated from the intestines, according to the statement of many medical practitioners; but these have only temporarily inhabited those viscera, and have originated from accidental introduction of their larvæ or ova.

The fourth chapter treats of the origin of worms in the human intestines, regarded in a pathological point of view. The author advances nothing on this subject that is satisfactory. It is thought that they will originate from a depravation of the nutritious matter contained in the intestines, depending on weakness of the intestines; or from more animal matter being present in them than can be absorbed; which matter, in virtue of its vitality and its tendency to form a whole and distinct being, then becomes organized. The more remote causes, the author thinks, are a state of languid vitality and inactivity of body; a moist, damp habitation, where moss and other vegetable matters are engendered; aliments of certain kinds, especially those of a very viscous and mucilaginous kind, or those which contain too great a proportion of nutritive matter, especially the abundant use of fat very easy of digestion, and milk. Those causes, he says, will explain how it is that verminous affections are sometimes endemic and epidemic. A low diet, and especially famine, he considers to be the most cruel enemy of worms; and he remarks, in support of this assertion, that the *lumbricoides* have been known to pass from the anus and mouth of persons keeping a very restricted diet, and when no medicine, or other substance, had been administered calculated to produce such an effect.

The next chapter treats of the diagnosis of worms in the human intestines. The author enumerates amongst the general signs of their presence, a change of the appearance of the countenance, the colour of which commonly becomes pale and of a leaden hue; but it is sometimes inordinately red, and, occasionally, in one cheek only. The eyes have lost their ordinary vivacity, and the lower eye-lids are bounded with a blue margin. The patient suffers a continual itching, or similar sensation, about the nose, which is also tumefied; and blood often escapes from the nostrils. There is sometimes pain in the head, and humming in the ears. The tongue is foul, and there is a great discharge of saliva from the mouth. The breathing is disturbed, especially when the patient is fasting; and there is often a short spasmodic cough. The appetite is irregular; sometimes it is quite lost, and sometimes it is ravenous. Nausea and inclination to vomit, and sometimes absolute vomiting, by which an aqueous fluid is brought from the stomach, takes place from time to time. There is pain in the bowels, often very severe, especially about the navel. The belly is tumefied and hard. The stools are mucous, and often streaked with blood. The urine is thick and whitish, as if pipe-clay or milk had been mingled with it; and the patients, when children, are disinclined to bodily exertions, and commonly peevish and irascible. The whole of these signs, the author adds, are but rarely present together; and there is often much difficulty in distinguishing whether those which do exist indicate worms or some other diseases: this difficulty exists especially in regard to the malady dependant on worms alone, and that constituting the prelude to hydrocephalus. Rosenstein points out the pleasing sensation experienced by the patient after having drank a glass of cold water, as an almost sure sign of the existence of worms; and it is probable that it is a circumstance of some importance in connection with the other signs above stated. Professor Brera says, that pains about the joints, similar to those of rheumatism, accompanied with dilatation of the pupil, with an inordinate and abundant secretion of saliva, and an inexpressible itching at the end of the nose, are, in children, and women of a weak habit of body, almost certain signs of the presence of worms in the intestines. Children, he also remarks, and individuals of a weak constitution and lax fibre, are the most liable to have worms. The ascarides vermiculares and lumbricoides are most common in children, whilst adults are mostly affected by the teniæ; and the development of worms is very frequent, he adds, in nervous fevers, and other (using his own terms) asthenic maladies; especially of the *tricocephali.*

On treating more particularly on this subject, Dr. Bremser remarks, that it is the *verminous disease*, or the causes of the formation of worms, rather than the presence of those animals, that should in most cases engage our chief attention; but he does not state any very precise or satisfactory signs of this verminous disorder. Its seat, he says, may be either in the organs of digestion and nutrition of the *first* or *second* order, that is to say, of digestion or of chylification; by means of which a matter is collected in the intestines, which, under favourable circumstances, will give origin to worms. It is this disorder, he thinks, which we have to treat in the generality of instances, and the cessation of it after the expulsion of worms from the intestines, is to be attributed, in the greater number of instances, to the remedies that have been employed; not to the mere disappearance of the worms, as is generally supposed.

Worms, of all the different species, are often found, too, to have been present in the intestines, without producing any symptoms by which their existence might have been suspected. Goetze and Rush even believed them to be necessary for the preservation of the health of children: an opinion which will not readily find partizans.

Although we are disposed to regard this view of the author in a favourable manner, we think he limits too narrowly the morbid effects which may be attributed to the presence of worms. It is not necessary for us to admit the doctrine of the spontaneous generation of worms in the intestines, in order that we may acknowledge the propriety of his therapeutical precepts; for, however they may originate, it seems sufficiently evident that a morbid state of the body is favourable, if not necessary, to their development, or their continued presence in the intestines. Persons of a robust habit are but very rarely affected with them; though, if they are derived from without, by means of ova received with the food, it is obvious that they must be exposed to them as much, if not more, than persons of a morbid habit of body, were not a healthy state of the intestines adverse to the development of the ova, or the existence of the living worm. If they originate by spontaneous generation, it is clear that the author's view must lead to measures of the most practical utility. It is worthy of remark, too, that persons, after having had the ordinary healthy state of their body destroyed by some accidental injury, will frequently then become affected with worms, (as Professor Brera, and several other physicians who oppose the doctrine of spontaneous generation, acknowledge,) although they had previously been free from them for a very long period.

Dr. Bremser denies the capability of worms to perforate the intestines, as many other physicians have supposed, on the grounds of observations which seem very favourable to such an opinion. We noticed, in a late Number of this Journal, (for January 1819,) some cases related by Mr. Gaultier-de-Claubry, in which a considerable number of ascarides had passed through openings in the parietes of the intestines; and there are many other analogous cases on record. Cases are mentioned in which ascarides were found in the gall-bladder, and it is said that they had entered it by perforating its parietes; but they are not related in such a way as to establish this proposition. In a case of the presence of those worms in that viscus, observed by M. Laennec, and related in a former volume of this Journal, (xv. p. 28,) it is obvious that they had passed into it by the ductus choledochus. But, the author argues that the perforations arose from other causes, and then the worms passed through them; and we recollect hearing Professor Chaussier (whose opinion on such a point of pathology is of the very first authority, as far as an opinion goes), express himself most inclined to admit this view of the matter. Rudolphi says that none of the worms in question are provided with organs qualified to pierce the coats of the intestinal tube when entire; whilst Jacopi asserts that the head of the ascaris lumbricoides is qualified to effect it, by means of the mechanism we mentioned when describing that animal.

Professor Brera, in his account of the symptoms especially dependant on the presence of the tænia, says that this worm sometimes fastens itself to the intestines by its head, and causes very violent pains in the belly, often productive of convulsions. The tricocephali, having no biting organ, produce, he thinks, less distressing symptoms, except when they exist in such numbers as to distend and obstruct the intestinal canal; a fatal instance of which obstruction is related by Lieutaud.

The presence of ascarides in the intestines, and especially in the rectum, is accompanied with a dull sense of irritation and a tedious insupportable itching, in addition to several of the general signs described on a former occasion. Professor Brera is disposed to attribute much of this irritation to want of the natural mucus of the intestines, which he supposes to be taken as food by the worms. Tenesmus and tumefaction of the anus are very commonly present with them.

The pains produced by the ascaris lumbricoides are most commonly felt about the navel: they are griping, like those of colic, and accompanied with a rumbling in the bowels. These circumstances, with the general signs of the presence of worms, mark pretty well the existence of this species. Cold water renders this worm immediately torpid; and hence, perhaps, the foundation of the diagnostic of Rosenstein.

It is not necessary to enumerate all the morbid effects of distant parts, as convulsions, &c. which occasionally arise from the presence of worms in the intestines, as they are not peculiar to this cause, being such as result from many other irritative causes acting on the same organs.

We should now pass on to the author's considerations on the several remedies which have been proposed or employed for the cure of verminous diseases; but it is necessary for us to defer our account of these until the next Number of this Journal.

---

*Observations sur le Coryza des Enfans à la Mamelle.* Par PIERRE RAYER, D.M.P. Médecin-adjoint du Quatrième Dispensaire de la Societé Philanthropique. 8vo. pp. 18. Paris, 1820.

THIS little tract contains some observations which every medical practitioner will acknowledge to be interesting, and calculated to explain the origin of some symptoms developed in the coryza of infants at the breast, that most experienced medical men will remember to have witnessed, though many may not have understood them so well as they probably will do after the perusal of the abstract we are about to give of this memoir.

The coryza of infants at the breast is characterized, Dr. Rayer states, by sneezing; tumefaction of the nose and eye-lids, and a shining appearance of the skin covering those parts; constant preservation of an open state of the mouth; a rather dry state of the lips and tongue; respiration accompanied by a nasal wheezing; impossibility of continuing to suck, though liquids put into the mouth are swallowed with facility: the infant takes the nipple in his mouth, but he has hardly made three or four suctions, when his respiration appears to be obstructed; his face becomes of a violet colour; he precipitately abandons the nipple, utters some cries, or is seized with a fit of severe coughing, which leaves him in a state of partial stupor. These accidents disappear in a short time, but are renewed whenever he again attempts to suck. This first stage of the disease lasts for four or five days, or thereabouts: it is followed by a secretion from the nasal cavities, the existence and quantity of which, at least in new-born children, it is not always easy to ascertain, because it either dries or falls into the pharynx when the infant lies horizontally on its back.

The following cases, with the author's remarks on them, will show more fully the nature and progress of this affection, and point out the measures for its treatment and cure.

"A well-formed, but weakly, male child, had continued to suck as well as such infants ordinarily do, from five hours after its birth until it was eight days old. At this time the infant seemed to be agitated; its mouth was kept half opened, and its lips and tongue were somewhat dry. Respiration was performed with difficulty, and accompanied with a sort of nasal sniffling, peculiar to coryza. It was in vain, many times in the course of this day, that attempts were made to get the infant to suck: as soon as he had made one or two suctions, his face became of a violet hue, he suddenly quitted the breast, and fell into a temporary state of partial torpor, after having had a fit of coughing.

"Persuaded that these accidents might depend on an unfavourable conformation of the nipple, or on some deleterious quality of her milk, the mother tried, with a sucking-bottle, to make the infant take by this means some cow's milk mixed with one-third of barley-water. The accidents above described were renewed, and I was sent for.

"The mother appeared to me to have all the qualities necessary for a good nurse; the nipple, especially, was well-formed. I passed my finger into the mouth of the infant; he gently sucked it, abandoned it, and began to cry. Wishing to be assured of the correctness of what had been stated to me, I requested the mother to put the nipple into the child's mouth: he had hardly made one or two suctions, when his face became of a violet colour; he cried violently, and fell into a state of partial stupor, after having suffered a violent fit of coughing.

"An attempt with the sucking-bottle, which I witnessed, was equally unsuccessful, and was accompanied or followed by the same phenomena.

"I then tried to make the infant take a spoonful of water sweetened with sugar: he swallowed it easily and almost with avidity. I gave him several others with the same success.

"The absence of any appearance of malformation of the tongue or of the mouth, the facility of deglutition, the occurrence of fits of coughing every time the infant attempted to suck, and the certainty that it had sucked very well on the preceding days; all these circumstances, joined with some particular symptoms which I observed, such as a shining appearance of the skin of the nose, with a tumefaction of this part and of the lower eye-lids, a nasal sniffling, the manner of respiration by the mouth, convinced me that all these accidents, and especially the impossibility of suction, were the results of inflammation of the mucous membrane of the nasal cavities. My directions to the attendants were, to make a fire in the room, which was very cold; to keep the infant warm and free from the influence of currents of air, to give it milk by a spoon, to foment its nostrils with a warm emollient decoction, and carefully remove the mucus collected in them; and I stated that *the infant would again be able to suck as soon as the state of the nasal cavities would permit it to breathe with the mouth closed.* Six days were sufficient to justify this prognosis.

"On the 20th of February, 1820, I was consulted by a woman of the lower class of persons, about a child, born at the full period and well-formed, six days old, which had refused to suck for the last eight-and-forty hours. It was supposed that the child was tongue-tied, and it was brought to me for the purpose of knowing whether or not this was the case.

"I was told that the mother was in good health, that her nipple was well-formed, and that she had plenty of milk. The child had sucked for the first four days like those infants do which are easily brought up.

"On examining the face of the child, I remarked a slight swelling of the eye-lids and nostrils, and a shining appearance of the skin covering those parts: the lips were dry; the mouth was half opened; the tongue and its frænum were well-formed.

"After having gently run the end of my little finger along the lips of the child, I passed it into his mouth; he seized it, and I felt his tongue applied to it. He made an inspiration as if he would suck; but he then immediately abandoned my finger, and began to cry in a shrill and plaintive manner. The woman who brought him to me said that, when he attempted to take the breast, he cried much more violently; and that he had sometimes such severe fits of coughing, followed by such a state of stupor, that they were alarmed for his life.

"Respiration was obstructed, and produced a sound, or nasal sniffling, peculiar to coryza. The child had taken with ease some spoonfuls of water sweetened with sugar, which I also gave it, that I might be assured of the way in which it swallowed. The whole of the circumstances of the case led me to regard it in the same way as the foregoing one; and I directed that the child should be fed with milk, and managed in a similar way. It lived for four days on cow's milk and barley-water alone. On the 26th and 27th of February it began to suck, and on the 28th it obtained thus the whole of its nourishment."

The author relates some other cases, all of which presented phenomena similar to those manifested in those just described, and which terminated favourably under similar management.

---

*Memoria sopra l'Allacciatura dell' Arterie.* Del Dottore Andrea Vacca Berlinghieri, Professore di Clinica Chirurgica nell' Imp. E. R. Università di Pisa; Cavaliere dell' Ordine del Merito sotto il Titolo di S. Giuseppe; e Membro di molte illustri Academie Europee. 8vo. pp. 60. Pisa, 1819.

Although this memoir does not contain any novel observations of remarkable importance, we consider that an abstract of it will prove interesting to a considerable proportion of our readers; for the recorded facts relating to the most essentially important circumstances of the subject of it, are yet far from being abundant; and diversities of opinion respecting the practical inferences that should be drawn from them, still exist amongst the most eminent surgeons of the present day.

The great advancement lately made by surgery in regard to the treatment of aneurism, and which all Europe freely acknowledges to be chiefly due to British surgeons, has made the question of the mode of tying the arteries, and the subsequent management of the ligature, of the most serious importance; and they have engaged much earnest attention from every surgeon who has actively exerted himself for the improvement of his art. Professor Vacca has entered the arena somewhat late, but not too late to acquire a share of the honour he so liberally appropriates to some of those who have preceded him in the discussion of those questions. Like most men who have exerted themselves with any advantage to science, he has not depended on the observations of others alone, where it was in his power to contemplate the elementary facts relating to the subject: he himself instituted a series of experiments on dogs, in order to furnish some original grounds for his reasonings. Those experiments are twenty-five in number, and were instituted according to the several views indicated by the different relations of the question above stated; that is, with different sorts of ligatures, and with diversities in the periods during which they were suffered to remain on the vessels. The history of these experiments cannot be abridged; we must, therefore, only occupy ourselves with his inferences from them, which appear to be precise and accurate; and the experiments seem to have been conducted with sufficient care. His first general conclusions are:

"1°. That the ligature of arteries producing obliteration of those vessels in the point tied, generally gives rise to the formation of clots of blood in the vessels, and to adhesion of their parietes.

"2°. That those two effects take place both from the ligature which divides the internal and middle coats of the vessels, and from that which simply maintains the inner coat in mutual contact; but that the ligature which produces a division of the coats, obliterates the vessel before that which does not divide them.

"3°. That the obliteration of an artery under the agency of a ligature does not take place according to invariable laws, although always produced by means of coagula of blood and adhesion, and is not effected in a constant determinate number of hours.

"4°. That the ulcerative process inseparably attendant on this species of ligature does not commence always at the same epoch; nor is it always completed in the same space of time.

"5°. That the removal of the ligature on the fourth day, does not arrest the ulcerative process, which, continuing to proceed, at length divides the artery.

"6°. That secondary hemorrhage is not a consequence of the ulcerative process, except when the coats of the artery, or other parts of the subject, are in a condition disposed to disease, or in a morbid state."

The first conclusion, the author properly remarks, admits of no doubt; but the second might appear disputable, if the results of the experiments of Scarpa opposed it: but he argues that the results of his experiments are not in opposition to those of the Professor of Pavia, and that they only differ in their opinions respecting them. The results of his experiments show that the ligature of an artery produces by the fourth, or even the third day, such a change in the vessel as opposes the passage of the blood through the tied point, in the great majority of cases. Scarpa had observed the same; and, as he on the third day found the circulation stopped at the tied point, as well in those instances where he had employed a round ligature as in those in which he had used one of tape, he concluded that obliteration of the artery was effected in the same time by one method as by the other: a conclusion which the author does not think correct, because, on removing the two kinds of ligature above mentioned, made on the same animal and on corresponding vessels, some hours after their application, he had occasion to observe that the obliteration was generally effected within the first few hours where the interior tunics of the vessel had been divided, whilst it was not completed until the third day when those parts were merely kept in contact without being divided. He therefore asserts that his experiments are not in opposition to those of Scarpa, and that the opinion of Jones and Travers is not erroneous in this respect.

The conclusion, he says, which seems to be in open contradiction with the results of the experiments of Scarpa, is the fifth; and this point, he thinks, merits being discussed with the greatest care.

Scarpa applied ligatures to the crural arteries of three sheep and a dog, removed the ligatures on the fourth day, killed the animals on the ninth day, and found the coats of the arteries undivided, though the cavity of the vessels was obliterated. He does not state whether or not any suppuration about the parts existed on the ninth day, nor whether or not the wounds were perfectly cicatrized.

On these facts Professor Vacca remarks, that the apparent opposition of the results just stated to those of his experiments, may perhaps be attributed to the diversity in the species of animal employed, or to some fallacious appearances, which had once led himself, and may also have led Scarpa, into error. At the same epoch (that of nine days), the artery in one of his experiments appeared to be continuous, although no doubt could be entertained of its having been divided by the ligature; for this had come away spontaneously, still maintaining its circular form, with the cylinder placed beneath the knot still enclosed in it. A tape was used in this experiment. The strong adhesion of the artery with the surrounding indurated cellular tissue, and the confusion of parts which thence resulted, prevented the matter being seen with much clearness. The author examines the other observations of Scarpa which appear to militate against the conclusion in question, and succeeds in showing that they are not sufficiently precise to present any forcible objections to his statement. He nevertheless acknowledges that the total division of the artery, although the ligature is removed on the fourth day, may not be without exceptions. The same external causes, he remarks, produce different effects in different individuals; and, he adds, "because it has never occurred to me to observe the obliteration of an artery on which a ligature had been kept for the space of only from four to six hours, have I reason to conclude that Jones, Travers, and Brodie, have asserted what is not true?"

Only two of his experiments are precisely applicable to the question alluded to in the passage just described, (that of the removal of ligature, in cases of the operation for aneurism, after it has been applied only a few hours, in order that the wound may unite by the first intention:) in one of these the ligature, (a round ligature composed of two threads,) tied as tight as possible, was removed from the crural artery of a dog four hours after its application. "The blood, for the space of a minute after its removal, did not pass the tied point; but it flowed through it manifestly after that time. Unfortunately, the dog was now claimed by his master, and could not be submitted to further researches." It is not certain, then, though it is probable, that the interior and middle coats of the vessel were divided by the ligature. In another experiment, where the ligature was a single thread, and tied as tight as possible, the ligature was removed when ten hours had elapsed from the time of its application: "the blood did not pass the tied part for the first two minutes, after this it traversed it freely." The dog was killed at the end of sixty hours. The internal and middle coats of the artery corresponding to the ligature were found to be divided. In other experiments, the ligature was removed either after a much longer or a much shorter period: or it had not been tied sufficiently tight to divide the interior coats of the artery, and therefore the results of them are not closely applicable to the question under consideration.

The sixth conclusion, the author also remarks, is not less correct than the others; for, in whatever way the vessels are tied in animals, cutting or not cutting a part of their parietes, removing at an early period the ligature, or leaving it to separate spontaneously, we never find secondary hemorrhage take place; and this happens, probably, because the animals submitted to such experiments are in a healthy state. These considerations, and the fact that some morbid state of the arteries has actually been witnessed in many cases of secondary hemorrhage, lead him to form the following inferences. "If then," he says, "facts and reasonings prove that the ulcerative process does not commence, under the agency of the ligature, always at the same epoch; that this process is established although the ligature be removed on the fourth day; that this process divides the artery; and that consecutive hemorrhage is generally the consequence of a morbid state of the parts, or of some unknown cause; we may conclude that the removal of the ligature on the fourth day will not prevent those serious accidents which it is desired to avoid. But, independent of this, let us see if the method of removing the ligature at an early period is capable of producing other advantages, or of giving origin to serious evils."

The author attributes the first employment of the temporary ligature to Mr. Travers, instead of Mr. Copland Hutchison. We shall not stop here to explain the way in which whatever merit may be due to the author of this practice, for the original attempt, was once transferred to Mr. Travers; but proceed to give Professor Vacca's opinion on its utility. It is unfavourable to the practice. After having adduced various pathological observations, in addition to those designated in the passages above cited, he concludes that "the removal of the ligature on the fourth day is a proceeding, in my opinion, condemned by reasoning and by experience; capable of producing but the most trivial advantages, whilst it may lead to evil consequences of the greatest importance." On this point there is, it seems, but little difference of opinion among surgeons; but, in the notions expressed in the following passage, he is at issue with English surgeons. Alluding to Scarpa, he says, "I entirely agree with him on the preference that should be given to the tape ligature joined with the cylinder, to the round ligature, since the somewhat more prompt union of the arterial parietes, obtained by means of division of the interior coats of the vessel, is an advantage of no value; and this method tends to produce a more prompt total division of the artery, and consequently consecutive hemorrhage:" and he concludes his memoir with remarking, that "new facts will perhaps throw greater light on this point of surgery; but, in the present state of the matter, I think myself authorized to conclude that we are not yet acquainted with the means of avoiding with certainty consecutive hemorrhage, which arises, probably, from some morbid state of our solids, or perhaps from a similar condition of the fluids; and that, finally, the only method of rendering it less frequent, is that of retarding as much as possible the separation of the ligature."

